# There is no “too small” for frequent workplace-based assessment: Differences between large and small residency programs in anesthesia when using a mobile application to assess EPAs

**DOI:** 10.3205/zma001709

**Published:** 2024-11-15

**Authors:** Tobias Tessmann, Adrian P. Marty, Daniel Stricker, Sören Huwendiek, Jan Breckwoldt

**Affiliations:** 1 University Hospital Zurich, Institute of Anaesthesiology, Zurich, Switzerland; 2 University Hospital Balgrist, Department of Anaesthesiology, Intensive Care Medicine and Pain Therapy, Zurich, Switzerland; 3 University of Bern, Institute for Medical Education, Bern, Switzerland

**Keywords:** competency-based education, CBME, WBA, EPA, entrustment, feedback, decision making, residency program, g theory, reliability, small business, SME, smartphone, mobile application

## Abstract

**Background:**

A competency-based education approach calls for frequent workplace-based assessments (WBA) of Entrustable Professional Activities (EPAs). While mobile applications increase the efficiency, it is not known how many assessments are required for reliable ratings and whether the concept can be implemented in all sizes of residency programs.

**Methods:**

Over 5 months, a mobile app was used to assess 10 different EPAs in daily clinical routine in Swiss anesthesia departments. The data from large residency programs was compared to those from smaller ones. We applied generalizability theory and decision studies to estimate the minimum number of assessments needed for reliable assessments.

**Results:**

From 28 residency programs, we included 3936 assessments by 306 supervisors for 295 residents. The median number of assessments per trainee was 8, with a median of 4 different EPAs assessed by 3 different supervisors. We found no statistically significant differences between large and small programs in the number of assessments per trainee, per supervisor, per EPA, the agreement between supervisors and trainees, and the number of feedback processes stimulated. The average “level of supervision” (LoS, scale from 1 to 5) recorded in larger programs was 3.2 (SD 0.5) compared to 2.7 (SD 0.4) (p<0.05). To achieve a g-coefficient >0.7, at least a random set of 3 different EPAs needed to be assessed, with each EPA rated at least 4 times by 4 different supervisors, resulting in a total of 12 assessments.

**Conclusion:**

Frequent WBAs of EPAs were feasible in large and small residency programs. We found no significant differences in the number of assessments performed. The minimum number of assessments required for a g-coefficient >0.7 was attainable in large and small residency programs.

## Zusammenfassung

**Hintergrund:**

Kompetenzbasierte Weiterbildung erfordert häufige arbeitsplatzbasierte Assessments (engl. Workplace-based Assessments, *WBAs*) von anvertraubaren professionellen Tätigkeiten (engl. Entrustable Professional Activities, *EPAs*). Mobile Apps erhöhen die Effizienz, allerdings ist es nicht bekannt, wie viele Assessments für zuverlässige Bewertungen erforderlich sind und ob das Konzept in Facharzt-Weiterbildungsstätten jeder Größe umgesetzt werden kann.

**Methoden:**

Über 5 Monate wurden mit einer Smartphone-App 10 verschiedene EPAs im klinischen Alltag in Schweizer Anästhesieabteilungen beurteilt. Die Daten von großen und kleineren Weiterbildungsstätten wurden verglichen. Mittels Generalisierbarkeitstheorie wurde eine Mindestanzahl an Assessments berechnet, die für zuverlässige Beurteilungen erforderlich sind.

**Ergebnisse:**

In 28 Weiterbildungsstätten wurden 3936 Assessments von 306 Supervidierenden für 295 Trainees erfasst. Die mediane Anzahl an Assessments pro Trainee lag bei 8, mit einem Median von 4 verschiedenen EPAs, die von 3 verschiedenen Supervidierenden beurteilt wurden. Wir fanden keine statistisch signifikanten Unterschiede zwischen großen und kleinen Weiterbildungsstätten in Bezug auf die Anzahl der Assessments pro Trainee, pro Supervisor*in, pro EPA, die Übereinstimmung zwischen Supervidierenden und Trainees oder die Anzahl der angeregten Feedbackprozesse. Der durchschnittliche Grad an Supervision (engl. *level of supervision, LoS*, Skala von 1 bis 5) lag bei größeren Programmen bei 3,2 (SD 0,5) im Vergleich zu 2,7 (SD 0,4) (p<0,05). Um einen g-Koeffizienten >0,7 zu erreichen, musste mindestens eine zufällige Auswahl von 3 verschiedenen EPAs bewertet werden, wobei jede EPA mindestens viermal von vier verschiedenen Supervidierenden bewertet werden musste, was insgesamt 12 Assessments ergab.

**Schlussfolgerung:**

Häufige WBAs von EPAs waren sowohl in großen als auch in kleinen Weiterbildungsstätten durchführbar. Wir fanden keine signifikanten Unterschiede in der Anzahl der durchgeführten Assessments. Die für einen g-Koeffizienten > 0,7 erforderliche Mindestanzahl von Assessments war in großen und kleinen Weiterbildungsstätten erreichbar.

## 1. Introduction

### 1.1. Competency-based medical education (CBME)

CBME and its implementation through the concept of Entrustable Professional Activities (EPAs) has increasing uptake in graduate medical education [1], [2]. An EPA can be seen as a frame through which one or more competencies can be observed during concise, specific tasks in clinical practice [3], [4]. The sum of all EPAs in a specialty represents its complete curriculum and thus all requested competences at a certain point of training. When combined into a portfolio, sets of complementary EPAs can represent important developmental stages [5] in a holistic, longitudinal approach. First published by Ten Cate 2005 [6], the concept of EPAs not only complemented and facilitated the rise of CBME, but also introduced the important aspect of trust and entrustment decisions for future performance. Evidence shows the use of entrustment-supervision scales rather than generic scores to have a greater clinical relevance, better inter-rater reliability and are easier to use [7], [8]. They do not provide a simple school grade, but an answer to the question: in a similar situation, how much supervision will my resident need next time?

To assess EPAs, a broad array of different workplace-based assessments (WBA) is used. Each WBA should not only serve as a tool for evaluation of trainees’ current status, but also support their future development through frequent feedback [9]. This concept of “assessment for learning” [10] has been introduced as one of the key components of a programmatic assessment approach. Furthermore, the results of these frequent WBAs should not be used for high-stake decisions like “pass/fail” of a residency program’s year. Contrarily, these should be made in so-called competence committees based on data from a variety of sources [11], [12], [13], [14].

To achieve more frequent and brief assessments, mobile applications have been introduced [15], [16], [17], [18], [19], [20], [21], [22] leading to grossly reduced times spent for a single assessment [23]. Still, a considerable number of assessments are necessary for reliable estimates of a trainee’s level of competency. Reliability and validity of a tool are closely related to its design and usage in the appropriate context [24], [25], [26], [27], [28], [29]. No matter for which purpose an assessment is carried out and to which decisions its result might contribute, they have to be based on a reliable, transparent and correct set of information. 

Usage of entrustment-supervision scales not only involves less assessor workload, but also reduces the necessary number of assessments by around 50% for Mini-CEX [7]. However, the required numbers for other WBA tools are spread over a wide range [24], [29], [30], [31], [32], [33], and the minimum number of assessments for an app-based WBA of EPAs has not been established yet. The minimum number of assessments could be dependent on a variety of factors, such as the number of different assessors, the interaction between supervisors and trainees, or the size of a residency program [4], [8], [25], [34].

### 1.2. Differences between large and small residency programs

Smaller healthcare institutions play a crucial role in healthcare access for everyone on a global scale, esp. in rural and urban under-served areas [35], [36]. Accordingly, high-quality medical education in smaller institutions is a necessity. Besides their structural conditions (number of trainees and supervisors, case-mix and -volume, available resources) [37], smaller institutions differ in respect to the educational culture [38]. There is no *better or worse* situation, but different opportunities: 

While one report found large residency programs benefit from their institutions’ better infrastructure [39], other authors did not report significant differences [40], or found superior opportunities in smaller institutions for developing clinical skills, better supervisors’ attitudes, and a higher sense of self-efficacy in undergraduate medical education [41], [42], [43]. The more specialized care in large institutions is weighed out by a more generalist and holistic approach in smaller ones. In addition, smaller residency programs might offer better chances for developing non-clinical skills such as resilience and autonomy, while at the same time also being more demanding in the same respect [44]. The more intimate atmosphere, long-lasting mentorships and less fluctuation of staff [45] could benefit the quality of training [46].

### 1.3. Analogy with non-healthcare sector

Outside the healthcare sector, it has also been shown that small- and medium-sized enterprises (SMEs) face their own needs, difficulties and characteristics concerning workplace-based education, resulting in a specific educational culture [47], [48], [49], [50]. It can be characterized on two dimensions: being a more integrated instead of a more formalized training, taking place in a more enabling than constraining learning environment [51], [52]. Acknowledging the different educational culture in SME led to superior training outcomes [53], [54]. As one review puts it: “strategies which match the way a small business learns are more successful than formal training” [55].

### 1.4. Research question 

Frequent WBAs of EPAs in residency programs of different sizes have not been studied yet. Gaining insight into the differences and similarities between smaller and larger programs could help to determine whether and how frequent assessments can be applied over the full range of residency programs. 

We therefore compared the usage and results of EPAs assessed with a mobile application between large and small residency programs in anaesthesiology and conducted a reliability analysis providing the minimally required number of assessments and assessors to achieve reliable ratings per trainee.

## 2. Methods

### 2.1. Participants and program categories

The education committee of the Swiss Society of Anesthesiology (SGAR-SSAR) invited all Swiss anesthesia departments with residency programs to participate (free of charge). Over 5 months in 2021 all recorded assessments were analysed. According to the national classification, we defined “large residency programs” as departments providing programs of 3.5 or 3 years (categories A1 and A2), and “small residency programs” as those with programs of 1 or 2 years (categories C and B). The large departments host faculty of >50 persons and belong to hospitals which offer highest-level care in a variety of specialties and usually serve as referral hospitals for their respective region. The smaller departments represent the spectrum of primary and secondary healthcare and feature most of the characteristics used for classification above [37]. For completing residency training, a minimal time of one year at a “small” institution is required in Switzerland. 

### 2.2. Mobile application and EPAs

The mobile application “prEPAred” (*precisionED Ltd, Wollerau, Switzerland*) allows assessing EPAs close to real-time with minimal interruption of daily workflow [23]. The trainee initiates the process by selecting the EPA performed. Then both trainee and supervisor, independently assess the complexity of the EPA (“simple” vs. “complex”) as well as the recommended LoS. Through comparison of the assessments a feedback conversation is stimulated which is optionally guided by the app using structured feedback instructions. Each assessment datapoint is stored in the trainee’s ePortfolio which the trainee may share with the supervisors [23].

The introduction of the prEPAred app was accompanied with information material such as instructional videos about CBME, EPAs and the app. 

For the trial period, we suggested ten common EPAs (see table 1 (Tab. 1), left column), largely resembling the catalogue of first to second year of the national aesthesia training curriculum [56]. Nevertheless, the entire EPA catalogue was available to choose from in the app (so as to provide the trainees and supervisors with the most benefit for individual education). The levels of supervision ranged from 1 (=observe only) to 5 (=supervise others) [57], [58], [59], as shown in table 2 (Tab. 2).

### 2.3. Statistics and g theory model

As the primary endpoint, we compared numbers of assessments per trainee and per supervisor. Secondary endpoints were differences in single EPAs assessed, the respective ratings (as defined through the supervisor-assessed level of supervision (LoS)), agreement between trainees and supervisors on the LoS, incidence of feedback stimulated by the assessment, and finally, a reliability analysis providing the minimally required number of assessments and assessors to achieve reliable ratings per trainee in a theoretical model (by using g-theory and d-studies).

For the general comparison of supervisors’ ratings of larger and smaller residency programs Wilcoxon U-tests were performed. Bonferroni correction of the alpha error was applied for multiple testing.

G theory is routinely used for the evaluation of assessment tools by trying to quantify and attribute the amounts of variance for individual facets and their interactions [27], [29].

To achieve a balanced dataset for g theory calculation we selected out of all possible trainee/supervisor combinations a sample of 20 trainees, who had completed sets of 3 EPAs with 4 assessments made by 4 different supervisors each. This decision was made a priori for statistical analysis based on the literature findings regarding minimal numbers for similar assessment tools [29], [30], [31], [32], [33]. 16 of the 20 trainees underwent training in large residency programs, the other 4 in small residency programs, reflecting the proportions of the overall sample.

We calculated the impact of the single EPA, the rating (LoS needed according to the supervisor) and the residency program on the variance of the trainees’ assessments. The g theory model was: epa x assessment x (trainee : residency program). 

The respective variance components were used in D-studies to determine the minimal number of assessments and assessors, respectively. 

A g study (epa x assessment x trainee) and D-studies were also calculated separately for larger and smaller residency programs to compare them against each other and against the overall calculation. To control for and quantify possible differences and interaction effects, a 3-way split-plot ANOVA was performed with category of residency program (large vs. small) as between group factor and EPA (3 levels) as well as supervisor (4 levels) as within subject’s factors. Dependent variable was the supervisors’ LoS. Only the 20 trainees that were eligible for the g study analysis were considered for this analysis.

G theory and d studies were calculated using G_String A Windows Wrapper for urGENOVA [60]. All other statistical computings were done with SPSS for Windows version 28 (IBM, Armonk, NY, USA). 

### 2.4. Data safety and ethics

Due to the strict data privacy policy of precisionED Ltd. and to avoid any concerns and hesitation to participate, no further data (e.g., age, gender, years of training) was gathered. The study was granted exemption by the Ethical Committee of the Canton of Zurich/BASEC-Nr. Req-2019-00242 (March 21, 2019).

## 3. Results

### 3.1. Descriptive summary of data

Between April 1^st^ and August 31^st^ 2021, 306 supervisors and 295 trainees used the prEPAred app to record 3936 assessments. We excluded 93 incomplete assessments (by the trainee *and* the supervisor, 2.3% of the total sample) from the dataset beforehand. In addition, 197 partially incomplete assessments (either by the trainee, or the supervisor, 4.5% of the total sample) were excluded from the respective analyses.

Overall, 28 of the 53 residency programs (52.8%) in Switzerland participated, of which 15 were large programs and 13 were small. Around three quarters of the supervisors and trainees worked in large programs and, accordingly, three quarters of all assessments were recorded there.

Of 722 registered anesthesia residents in Switzerland in 2021 [61], 41% participated in the study. In the participating residency programs, 496 residents were registered, resulting in a 69% participation rate (58% in large, 78% in small residency programs).

The median number of assessments per trainee was 8, the median number of different EPAs assessed was 4, by a median of 3 different supervisors. In 2261 (57%) of all assessments, a feedback process was initiated and in 2165 (96%) of those, a learning goal was documented (see table 3 (Tab. 3)).

### 3.2. Comparison of large and small residency programs’ results

Overall, we found no relevant differences between large and small programs. In specific, we found no significant difference for the number of assessments, trainees, supervisors, average number of supervisors per trainee, average number of assessments per trainee or per supervisor, or incidence of feedback stimulated (see table 3 (Tab. 3)). We also did not find statistically significant differences regarding the agreement between the supervisor’s and trainee’s rating on the required LoS and the task complexity. Furthermore, the distribution of single EPAs assessed was similar between the groups (see table 1 (Tab. 1)), as was the average rating of complexity. 

As one statistical difference (p<0.01) the average supervisors’ LoS ratings, were higher in large programs for almost all EPAs, with an average of 0.5 points more. The other significant difference between the groups was the higher amount of “fixed pairs” in smaller residency programs, cf. to paragraph 3.4. 

### 3.3. Details on EPAs and resp. ratings

“Induction of anesthesia” and “tracheal intubation” were the two most frequently assessed EPAs, making up for one third of all assessments. The remaining assessments were evenly distributed between the other EPAs (cf. to table 1 (Tab. 1)).

There were no differences in LoS ratings between single EPAs, with an overall average of 3.11 (SD 0.32), i.e., indirect supervision is required. The trainees’ self-assessed LoS for all EPAs was slightly lower than the supervisors’ ratings, but no statistically significant difference could be shown. The same holds true for the ratings of complexity, showing a mean of around one third of all assessed events being classified as complex.

### 3.4. “Fixed pairs” and “enthusiasts”

In both sizes of programs we found fixed pairs (i.e., one trainee collaborating with the same supervisor for 4 or more assessments). Of these 317 pairs, 250 (22.4% of 1116) resp. 67 (38.3% of 175) worked in large resp. small programs. The difference was statistically significant (Chi^2^(1)=20.604, p<0.001). These pairs accounted for 1843 (46.8% of total) of all assessments: 1381 (44.7%) in large and 462 (52.7%) of them in small programs (Chi^2^(1)=24.68; p<0.001). 

Within the trainees and the supervisors the number of assessments highly varied, with single individuals having recorded more than 200 assessments (2 supervisors and 1 trainee from large and 1 resp. 2 from small programs). 3 of the 15 large and 5 of the 13 small programs returned only a few assessments (<34 assessments).

### 3.5. G theory

The results for the g theory models are shown in table 4 (Tab. 4), yielding a robust result of phi >0.7 with the chosen combination of assessments/EPAs/supervisors while attributing 21% of variance to the facet of interest (the trainee), nested in residency program (as dictated by study design). For the 20 trainees that entered the g theory analysis, the 3-way ANOVA revealed no significant differences in entrustability rating between programs, EPAs or supervisors, nor were there any significant interactions.

**Figure 1:**
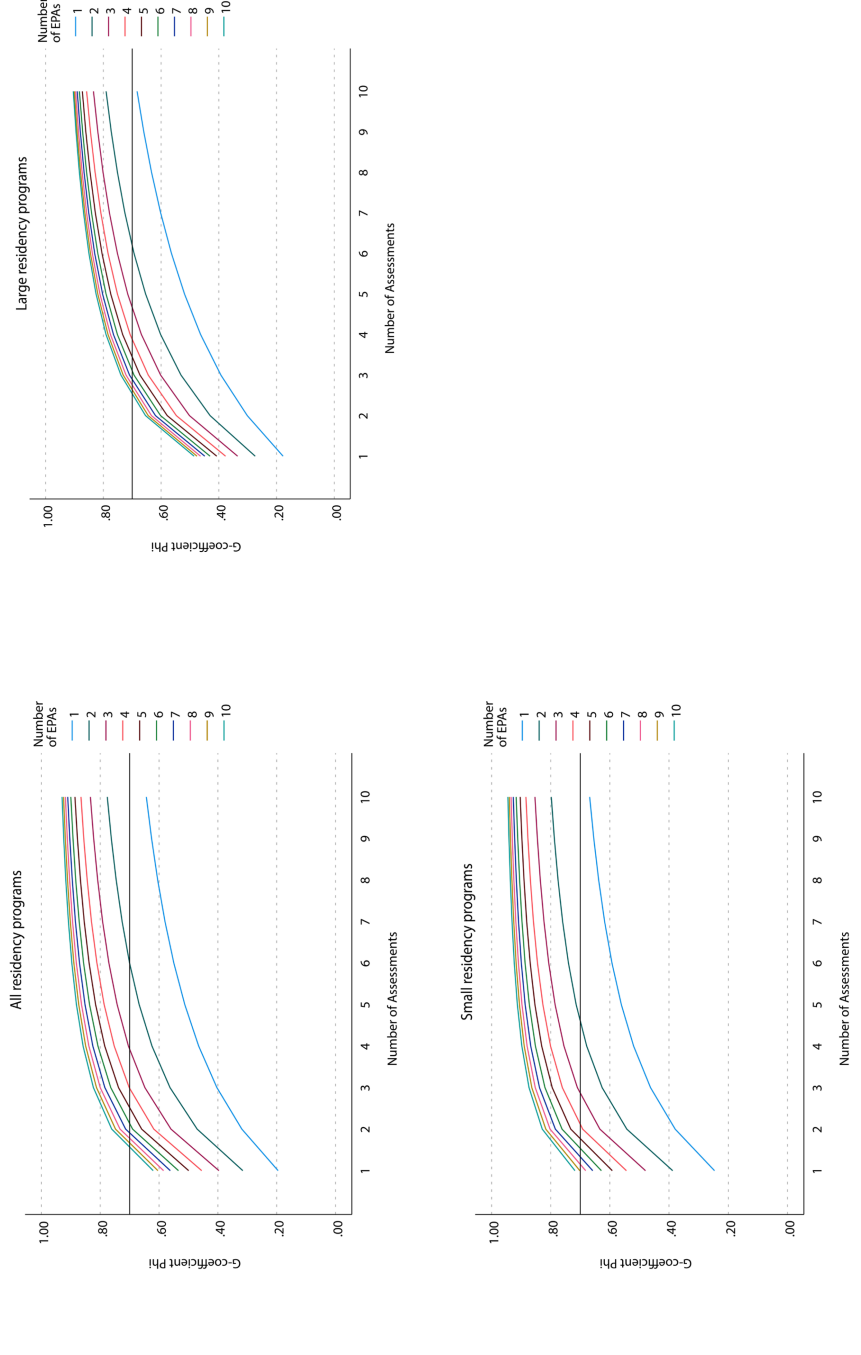
D studies to table 4

All respective D studies are shown graphically in figure 1 (Fig. 1). The main result can be found in figure 1 (Fig. 1) (dark red line): theoretically sufficient (phi coefficient of 0.7) assessments can be expected with at least a random set of 3 different EPAs out of 10, with each EPA rated at least 4 times by 4 different supervisors, resulting in a total of 12 assessments. 

A phi coefficient of 0.8, which can be interpreted as extraordinarily robust measurement tool, can be expected when assessing random sets of 6 EPAs with 4 different supervisors (again, totalling 12 assessments).

## 4. Discussion

### 4.1. Comparison of large and small residency programs

Overall, we found a broad participation generating a sufficient number of assessments in both large and small residency programs. Considering the voluntary participation, there was a substantial interest in the topic as reflected by a participation rate of 69%, which was even higher in smaller programs than in large ones. This indicates a high motivation to be part in new educational developments and confirms previous results [17], [23], [62]. The very few incomplete data sets might indicate the app’s ease of use in daily routine.

In contrast to previous results both in medical education and in the non-healthcare sector, we found no relevant difference between the two sizes of programs. This applies to the number of assessments acquired, the feedback initiated and the distribution over trainees and EPAs, indicating that frequent workplace-based assessments using EPAs are feasible in both settings. 

The significantly higher LoS ratings in large programs (indicating less need for supervision) can be due to a variety of reasons: differences in daily routine, expertise, or educational opportunities. For example, in large institutions with longer lasting and higher-risk surgery, arterial cannulation is likely more routine. This could explain why the LoS for this EPA was 0.7 points higher. 

On the other hand, there might be a stronger “educational alliance” [63], [64] between trainees and supervisors in small programs, with a higher frequency of one-on-one teaching [41], [43] and less fluctuation of staff [45]. Our finding of a significantly higher proportional number of fixed pairs and assessments acquired by them in small compared to large programs further supports this assumption. Finally, as in non-healthcare industries [49], this could result in a different educational culture in small programs, in which the perception of required and provided supervision might be higher. This could shift the scale of LoS, leading to closer supervision in comparable instances of an EPA. In the end, each individual rating of the LoS by a supervisor is subjective and influenced by the interpersonal relationship [65], [66], [67].

### 4.2. Agreement in LoS

In a previous study using the prEPAred app [23], in 35.6% of assessments there was a divergence in assessed LoS between trainee and supervisor. Similarly, we found this to be the case in 33% of cases and also showed the same clear trend for the trainees to rate themselves slightly less autonomous than their supervisors did. 

### 4.3. Required number of assessments and supervisors

We calculated that at least 12 assessments of 3 different EPAs by 4 different supervisors are necessary for a reliable rating in theory (as these ratings had no practical relevance in our setting). The g coefficients >0.7 are in compliance with recommendations for assessments like OSCEs or other high-quality assessments [26], [68]. We even observed higher phi values for small programs, indicating that small programs might at least be comparable to larger ones. Therefore, the EPA assessments via the app used in this study yielded reliable results for random sets of 3 different EPAs out of 10. 

When used on a broad, regular basis, it is likely that most residents will quickly achieve sets of more than 3 out of these 10 EPAs, as all of them are expected to be mastered by the end of the second year of residency [56]. Thus, a typical portfolio will display a variety of assessments and support the supervisors’ decisions, which clinical tasks can be entrusted to residents. Frequent observations and feedback during daily routine can be expected to improve training as proposed in a programmatic assessment approach. We advise such frequent assessments for improving residence training regardless of the size of the program.

### 4.4. Limitations

A potential source of bias is the voluntary participation of both supervisors and trainees, resulting in a non-representative sample for both groups. Further, it remains open what the practical implications are based on a reliable rating of random sets of 3 different EPAs out of 10. Nonetheless, this data might provide insights for future practice implementation. Investigating the applicability of the findings to mandatory conditions is pivotal.

Data on potential confounders such as gender, age, years of training, etc. was not available due to the strict data privacy policies as stated before. As these factors are likely to have an impact on educational processes, results might have been different if corrected for the confounders. However, this study compared programs of different sizes, and the distribution of confounders is likely to be similar, even more, as rotations in small and large programs are mandatory in Swiss anaesthesiology training.

## 5. Conclusion

Frequent WBAs of EPAs using a mobile application showed to be reliable in both large and small residency programs. We found no relevant differences between the two sizes of programs regarding the numbers and distribution of assessments performed. G theory and D studies analyses confirmed that the minimum number of assessments for a g-coefficient >0.7 can be reached in both program sizes. Therefore, the support of competency-based education through mobile applications to assess EPAs appears suitable in large and small residency programs in anaesthesia. 

## Authors’ ORCIDs

- Tobias Tessmann: [0000-0002-4687-7452]
- Adrian P. Marty: [0000-0003-3452-9730]
- Daniel Stricker: [0000-0002-7722-0293]
- Sören Huwendiek: [0000-0001-6116-9633]
- Jan Breckwoldt: [0000-0003-1716-1970]

## Competing interests

APM is member of the educational committee of SGAR-SSAR and member of the EPA Committee of the Swiss Institute for Medical Education (SIME). With grant money from the University of Zurich’s “Competitive Teaching Grant” and a grant from the SIME, a first functional prototype was developed by an external software company in 2019. In fall 2020, APM founded a company (precisionED Ltd) to rebuild the App from scratch and to provide a sustainable high-quality assessment system. precisionED holds all intellectual property rights and guarantees state-of-the art protection of any data by complying with GDPR-standards.

SH is member of the EPA Committee of the SIME. JB is member of the EPA Committee and co-chair of the “teach the teacher” program of the SIME.

## Figures and Tables

**Table 1:**
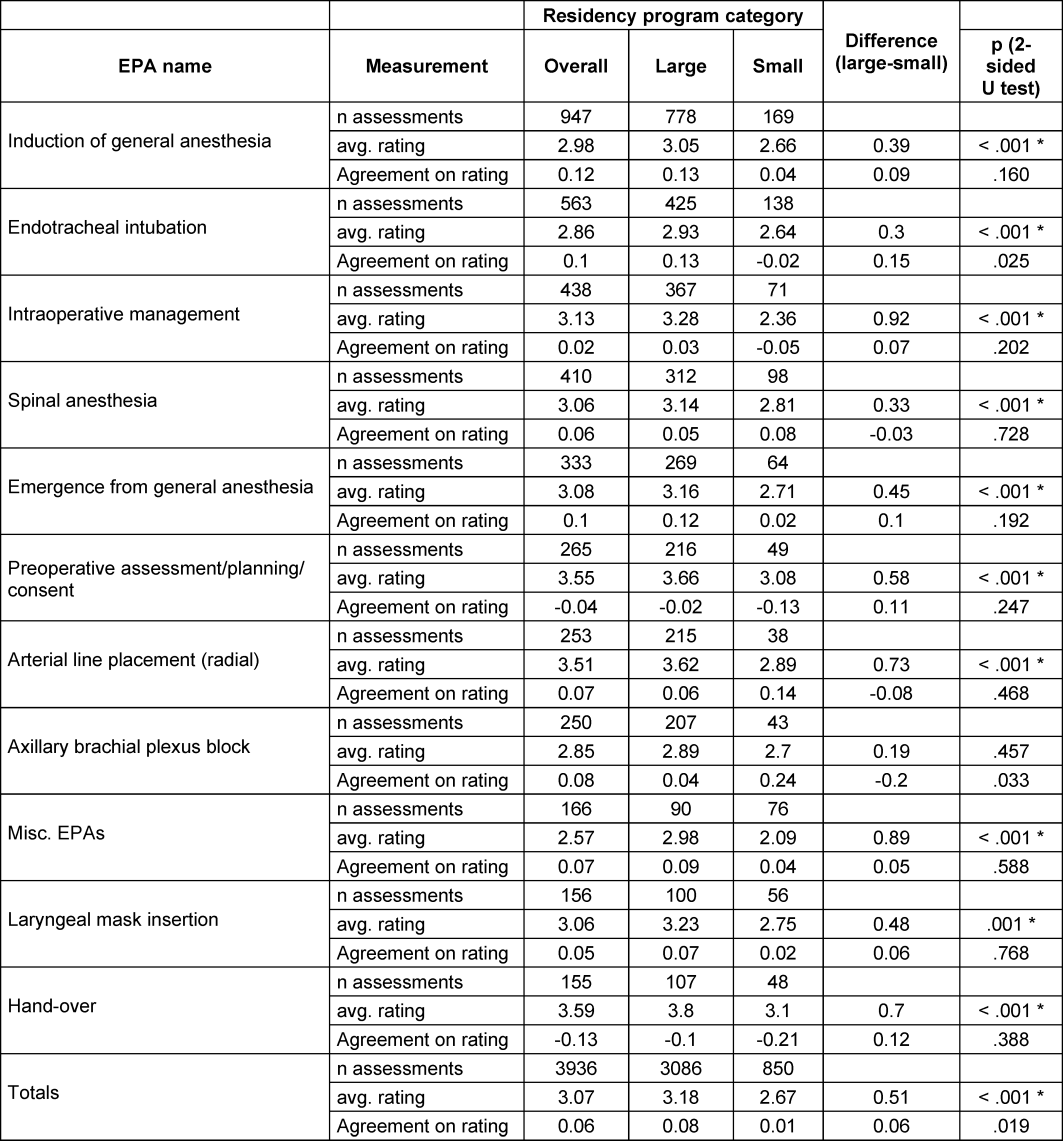
Single EPAs with number of assessments and average ratings per category, in descending order of total n

**Table 2:**
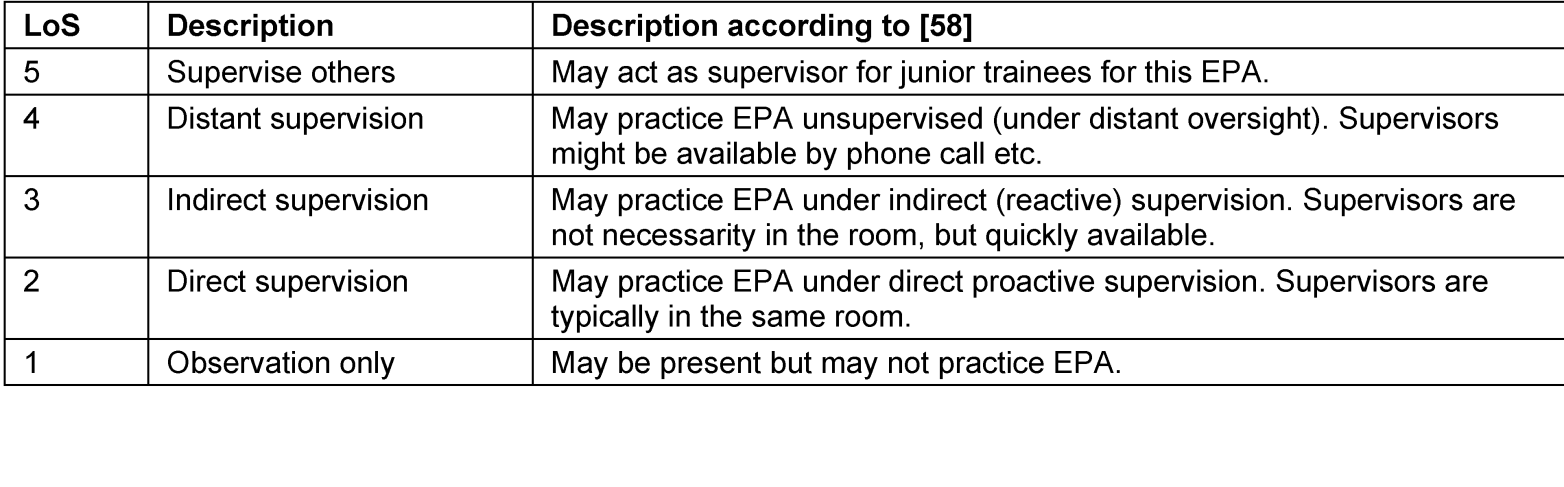
Description of the entrustment-supervision scale

**Table 3:**
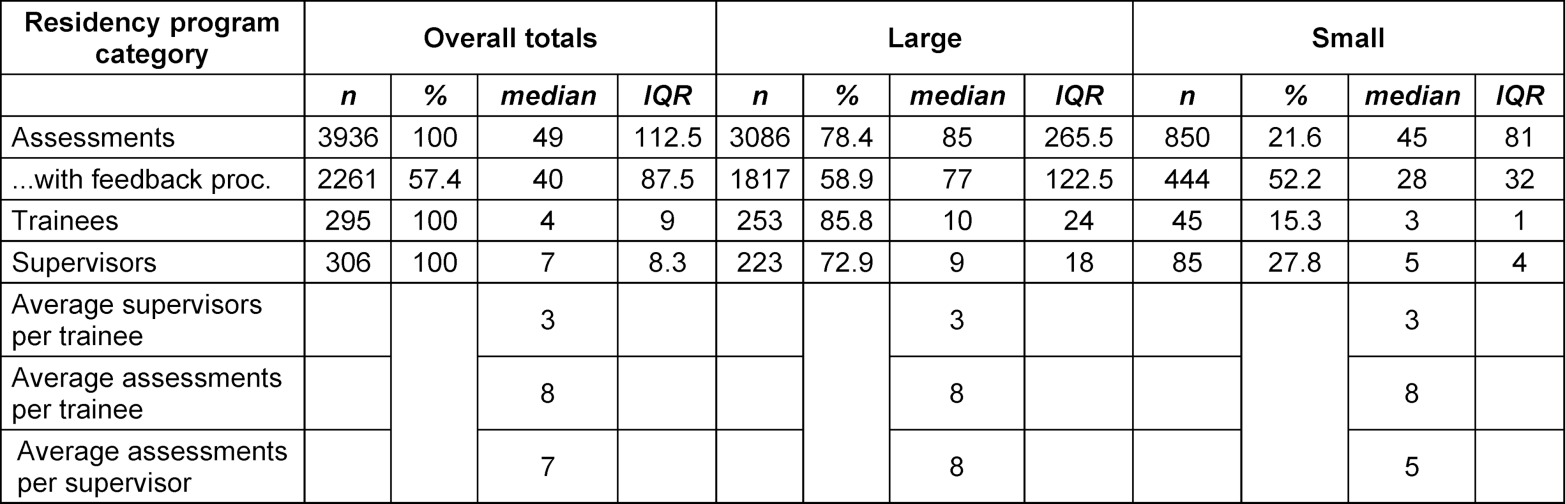
Number of assessments, trainees, supervisors and resp. averages per category

**Table 4:**
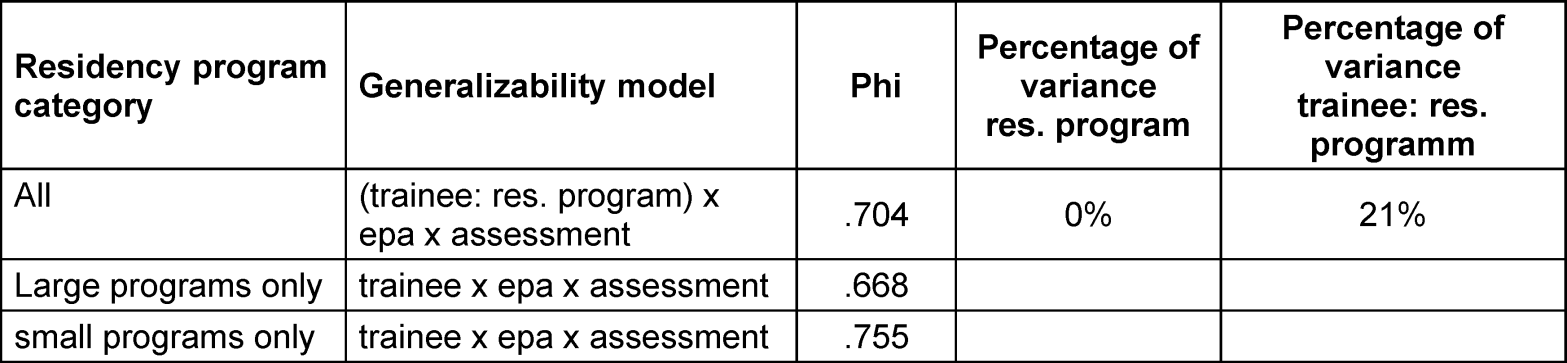
G theory results for 3 EPAs, each assessed 4 times by 4 different supervisors
